# Scientists versus Regulators: Precaution, Novelty & Regulatory Oversight as Predictors of Perceived Risks of Engineered Nanomaterials

**DOI:** 10.1371/journal.pone.0106365

**Published:** 2014-09-15

**Authors:** Christian E. H. Beaudrie, Terre Satterfield, Milind Kandlikar, Barbara H. Harthorn

**Affiliations:** 1 Institute for Resources, Environment and Sustainability, University of British Columbia, Vancouver, British Columbia, Canada; 2 Compass Resource Management Ltd, Vancouver, British Columbia, Canada; 3 Liu Institute for Global Issues, University of British Columbia, Vancouver, British Columbia, Canada; 4 National Science Foundation (NSF) Centre for Nanotechnology in Society, University of California Santa Barbara, Santa Barbara, California, United States of America; US Army Engineer Research and Development Center, United States of America

## Abstract

Engineered nanoscale materials (ENMs) present a difficult challenge for risk assessors and regulators. Continuing uncertainty about the potential risks of ENMs means that expert opinion will play an important role in the design of policies to minimize harmful implications while supporting innovation. This research aims to shed light on the views of ‘nano experts’ to understand which nanomaterials or applications are regarded as more risky than others, to characterize the differences in risk perceptions between expert groups, and to evaluate the factors that drive these perceptions. Our analysis draws from a web-survey (N = 404) of three groups of US and Canadian experts: nano-scientists and engineers, nano-environmental health and safety scientists, and regulatory scientists and decision-makers. Significant differences in risk perceptions were found across expert groups; differences found to be driven by underlying attitudes and perceptions characteristic of each group. Nano-scientists and engineers at the upstream end of the nanomaterial life cycle perceived the lowest levels of risk, while those who are responsible for assessing and regulating risks at the downstream end perceived the greatest risk. Perceived novelty of nanomaterial risks, differing preferences for regulation (i.e. the use of precaution versus voluntary or market-based approaches), and perceptions of the risk of technologies in general predicted variation in experts' judgments of nanotechnology risks. Our findings underscore the importance of involving a diverse selection of experts, particularly those with expertise at different stages along the nanomaterial lifecycle, during policy development.

## Introduction

Rapid advances in promising new nanotechnologies have been accompanied by mounting concerns over their human health and environmental risks – concerns that are exacerbated by the uncertainties inherent in this still-emerging domain [1]. Despite growing support for environment, health, and safety (EHS) research [2], decision makers in industry and government are in the very early stages of understanding and managing potential risks. Primary to regulatory conundrums is the question of whether and by whom nanotechnologies are seen as novel and as posing new kinds of risk, and whether current regulatory approaches are suitable for managing these risks [3], [4]. Some have argued that risks from engineered nanoscale technologies are not novel [5]; whereas policy analysts have found gaps in existing regulations and have identified numerous challenges for risk assessment. These include a high degree of scientific uncertainty, a paucity of nanomaterial risk data, and a lack of nano-specific risk assessment tools [1], [4], [6], [7]. The result is that regulatory agencies may be ill prepared for assessing and managing risks from emerging nanotechnologies [8]. Given these challenges, expert opinion will play an important role in the formulation of policies and programs to address nanomaterial risks [9].

Among those well situated to consider questions of risk and regulation are experts within the sector, including basic scientists and engineers, risk assessors and toxicologists, and those responsible for regulation of nanomaterials and products. Little is known, however, about how these different groups of experts view nanomaterial risks, and what drives those differences. This study examines experts' views of the risks posed by nanotechnologies, the approaches to regulation that experts' deem most suitable, whether perceptions of nanomaterials as novel influence their perceptions of risk, and how their perceptions vary given the particular ‘class’ of expertise to which study participants belong.

### Risk and Regulation

Experts' perceptions of risk have been studied in a number of domains, including genetically modified organisms [10], [11], chemicals and toxics [12]–[15], and ecological risks [16], [17]. This earlier work generally finds disciplinary field (e.g., physical versus biological sciences) [11], [14], institutional affiliation (e.g., university versus industry scientists) [13], [18], demographic position (e.g., gender, age, etc.) [11], [13], [18], [19], and/or social-political values (e.g., social or economic conservatism) [14], [20], [21] to be strongly predictive of perceived risk (regardless of the technological domain examined). In the nanotechnology case, a few recent studies have begun to identify factors underpinning risk judgments among nanoscientists. Besley et al. found that experts perceived different oversight needs and support as a function of their reported disciplinary field [22]. Similarly, Ho et al. found gender and trust (*in scientists and/or government*) to be predictive of perceived risk [23], while Siegrist et al. found trust to be a significant driver of risk perceptions [24]. Several studies have explored factors driving experts' *support* for nano regulation and perceived *adequacy* of current regulations for the nanotechnology case–as both discrete questions and as variables that correlate with risk judgments. Corley et al. found gender, discipline, and socio-political values to be predictive of *support* for nano regulation [25], while gender was a significant driver of perceived *adequacy* of regulations [26]. Further, experts' support for regulation was found to correlate positively with perceived risk [22], [25], [26], while perceived adequacy of existing regulations was found to correlate inversely with nanotechnology risks [22], [26]. These findings suggest that a combination of factors: gender, fields of expertise, and opinions about the risk object as well as the existing regulatory regime are all correlated with perceived risks from nanomaterials.

Critically what we do not know, given the shortcomings of existing regulation described above, is what kinds of regulatory approaches experts support or prefer, and how those preferences correspond to their risk perceptions. Recognizing that governance of emerging technologies can involve far more than top-down government regulation [27], a number of alternative policy and governance options could be exercised to manage this new and ubiquitous class of materials. These include voluntary or self-regulation by scientists and industry actors [28], market-based approaches wherein market signals and consumer choices modulate risk [29], and government approaches to regulation that provision information and operate using the principle of precaution [30].

In addition to our limited understanding of expert preferences for approaches to governance, there remains a dearth of research exploring experts' conceptualizations of the novelty of nanomaterial properties, and how those conceptualizations drive their overall perceptions of risk. [An exception is Powell (2007) [5], who interviewed a group of scientists (n = 20) and found novelty to be important to the way in which they framed or discussed risk]. Thus, experts' conviction about the novelty of nanomaterials remains a relatively uncharacterized phenomenon, and an untested driver of expert opinion. Similarly, while studies comparing evaluations of risk given a list of hazards or risk objects are somewhat common, no studies that we could find in the nanotechnology domain have tested whether perceived risk across a collection of technologies (new and old) is *predictive* of risk perceptions for a particular technology under study, in this case, nanotechnologies.

Finally, virtually all expert studies to date have focused on investigating the drivers of perceived benefit and risk *in reference to* or *within* particular expert groups [24], [31]. No studies have been conducted, however, which systematically classify and sample experts based on their specific role in 1) developing materials versus 2) studying their toxicological behaviour versus 3) assessing and managing their risks. Further, while interview-based studies have begun to characterize the opinions and perceptions of various nano expert groups [5], [32], several claims, such as the variation in novelty perceptions, have not been well substantiated quantitatively. Given the diversity of expertise involved in the nanotechnology enterprise, from ‘upstream’ researchers to ‘downstream’ risk assessors and decision-makers [5], it is important to understand how the perspectives of different expert groups vary with respect to the riskiness of technologies in general, conceptions of the novelty of nanotechnologies, and preferences for regulatory approaches.

### Hypotheses

Given existing research, this study examines expert perceptions of nanotechnology risks as linked to perceived novelty of nanomaterial characteristics, perceived risks from technologies in general, and preferred approaches to regulation. We also operationalize expertise in reference to three distinct groups: nano-scientists and engineers (NSE), nano-environmental health and safety scientists (e.g., toxicologists) (NEHS), and nano-regulators including those who assess and manage risks (NREG). Based on previous findings, which suggest that expert perceptions of risks and benefits vary across research and development domains [22], [25], and that each of these domains suggest different investigatory responsibilities and interests viz. nanotechnologies, our main hypothesis is as follows:

1. Nanotechnology experts working on *research and development* versus *EHS implications* versus *risk regulation* will differ significantly on their perceptions of benefits and risks from nanotechnologies.

Second, given debates about whether nanotechnologies are new or different from existing technologies, and given preliminary evidence that variation in the perceived novelty of nanotechnologies is evident across experts [32], and that perceived novelty of nanomaterial characteristics is linked with perceived risks [5], we propose two additional hypotheses:

2a Experts who see nanotechnology *benefits* as novel (i.e., as a new class of materials or objects with novel properties) will see less overall risk from nanotechnologies compared to those who see nanotechnology benefits as *not* new (i.e., as little different from their bulk form); and
2b Experts who see nanotechnology *risks* as novel (i.e., as a new class of materials or objects with new risks) will see more overall risk from nanotechnologies compared to those who see nanotechnology risks as *not* new;

Third, given earlier studies of expert perceptions of technologies' risks and benefits (i.e., those explored in non-nanotechnology domains such as chemical, biotech, or ecological risks) [10], [13], [17], [18], we also expect that:

3 Experts who assign higher levels of perceived risk overall (that is, across other technological domains such as nuclear power and GM foods) will see more risk from nanotechnologies as well, versus those who see less risk from other studied technologies.

Finally, given recent findings that experts' *support* for regulation [25], [26] and perceived *adequacy* of regulation [22], [26] are correlated with nanotechnology risk perceptions, we propose a fourth hypothesis:

4 Experts who prefer more government regulation (of nanotechnologies) and a more precautionary approach to risk management will see greater risk from nanotechnologies compared to those who view regulations as adequate, and who prefer market-based approaches to risk management.

## Methods

This research was conducted under the approval of the Behavioural Research Ethics Board, University of British Columbia, and the Institutional Review Board, University of California Santa Barbara. Written informed consent was obtained from all survey respondents. The data reported here were collected through a web-based survey (N = 404), designed to assess US & Canadian nanotechnology experts' perceptions of risks and regulation. The survey was conducted by the University of California Santa Barbara Social Science Survey Center for the UCSB Center for Nanotechnology in Society between June 2nd and November 8th, 2010. To construct the sample frame, we compiled names and detailed contact information for 2,100 experts within three pools of US and Canadian experts: nano scientists and engineers (NSE), nano-EHS scientists and toxicologists (NEHS), and scientists and regulators in government agencies (NREG). Subjects were contacted by email in a three-step process, including initial contact and two reminders at two-week intervals. Respondents received an ‘A’ or ‘B’ version of the survey at random, where the wording of several survey questions were modified to reverse the meaning of the question. Questions with alternate wording were reversed-coded during analysis to enable direct comparison of responses. Where appropriate the sequence of questions was also varied to minimize order effects.

For the NSE group, experts were selected using a rigorous sampling design, based on a bibliometric methodology developed by Porter et al. [33] using nanotechnology publications identified through ISI Web Of Science. We excluded papers with the following terms to remove publications that would fall under our NEHS sampling strategy: toxic* or genotoxic* or ecotoxic* or (oxidative stress) or safety or pollution or (environmental health) or (human health) or (animal health) or (public health) or (occupational health). Results were limited to articles and review papers by authors in the US and Canada. 1,200 subjects were selected at random from a pool of over 5,700 first or corresponding authors who published five or more nanotechnology articles that were cited five or more times between 2000 and 2009 (a method utilized by Scheufele et al. (2007)), with at least one article newer than 2006. Database searches were conducted between August and September 2009.

NEHS experts were selected from first or corresponding authors of 1,600 articles entered into the International Council on Nanotechnology (ICON) Environment, Health and Safety Database between early 2007 and spring 2010. Due to the relatively small domain of nano EHS research, we could not apply the same rigorous NSE standard of selecting authors with five or more publications, and instead selected 500 experts at random from a list of over 1,600 authors. International contacts were removed from the list, and several authors listed with .gov email suffixes were cross-referenced with the NREG group for duplications, and removed from the NEHS group.

NREG experts were identified from nanotechnology conference attendance lists, referrals, and website searches of employees in nanotechnology groups in US and Canadian Federal Regulatory agencies (including EPA, OSHA, FDA, CPSC, Health Canada, Environment Canada) and within Federal research institutes (NIOSH, NIH, national labs), as well as US State regulatory agencies (including Massachusetts Department of Environmental Protection, New York Department of Environmental Conservation, California EPA, North Carolina Department of Environmental and Natural Resources, and Washington Department of Ecology). Contact information and agency affiliation were compiled for 400 NREG experts in spring 2010. A full list of agencies is available in List S1.

A total of 404 responses were analyzed, for an overall response rate of 23% (AAPOR RR-3: 23%). In total 254 participants specified their residence in the US, while 55 reside in Canada, and 95 did not disclose their country of residence, and so might belong to either country. All analyses include both US and Canadian participants unless otherwise specified. Individual group response rates were: NSE: N = 180, RR = 16%; NEHS: N = 121, RR = 33%; NREG: N = 103, RR = 32%. We believe the relatively low response rate of the NSE group is due to a large number of outdated mail and email addresses (our search criteria includes publications since 2000). Contacts may have moved institutions or changed email addresses since the date of publication, and therefore were not measured as ‘bounced’ or ‘out-of-scope’. Separate response rates for the US and Canadian groups were not possible since not all respondents indicated their country of residence in their survey responses. Statistics were calculated using the SPSS software package [34]. **Table 1** outlines a breakdown of demographic and domain of expertise variables across the three expert groups.

**Table 1:** Demographic and Domain of Expertise variables by expert group.

Variables	Category	NSE (N = 171)	NEHS (N = 143)	NREG (N = 110)
***Demographic Variables***				
Year of highest degree (*mean (SD))*		1990.1 (11.3)	1994.0 (10.4)	1992.3 (10.2)
Gender (% Male)		89.1%	60.2%	64.9%
Education	*PhD degree (or professional degree e.g. MD, DVM, Doc of Law)*	99.3%	98.9%	48.7%
	*Masters degree*	0.7%	0.0%	35.9%
	*Bachelors degree*	0.0%	1.1%	15.4%
***Domain Of Expertise Variables***				
Proportion of time working on nano (mean (SD))		0.64 (0.28)	0.57 (0.30)	0.34 (0.34)
Involvement in Research		99.3%	94.7%	43.6%
Affiliation	*Academic*	81.9%	89.4%	0.0%
	*Government*	8.0%	1.1%	97.4%
	*Other (private sector, NGO, or specified response)*	10.1%	9.6%	2.6%
Disciplinary Field	*Physical Sciences (only)*	85.0%	13.7%	6.4%
	*Biological, Environmental, and Health Sciences (only)*	6.4%	60.0%	50.0%
	*Policy, Management, and Social Sciences (only)*	0.7%	7.4%	17.9%
	*Phys and Bio Sciences (both indicated)*	7.9%	15.8%	7.7%
	*Bio and Policy (both indicated)*	0.0%	3.2%	16.7%

Notes: All values (except for ‘year of highest degree’ and ‘proportion of time working on nano’) indicate the distribution of respondents by group for each variable (out of a total of 100%). Figures for the ‘year of highest degree’ and ‘proportion of time working on nano’ scales indicate mean scores and standard deviations.

Questions used to examine the above hypotheses are detailed in each relevant findings section. In brief, however, we relied upon two relevant question sets: 1) Those addressing assessments of perceptions of nanotechnologies' newness or novelty, and their benefits, properties, and risks; and we elicited evaluations of uncertainty and the suitability of existing methods for testing risks. 2) A second set of questions looked at preferences for regulatory approaches, judgments about the suitability of existing regulations and tools for managing risks from technologies in general, and nanotechnologies in particular.

## Results

### Expert Variation in the Perceived Risks and Benefits of Nanotechnologies

Risk and benefit judgments across different expert groups (hypothesis 1) were assessed by 1) evaluating respondents' overall perceptions of nanotechnologies using a *5-point risks versus benefits* scale (detailed in the ‘Differences in Overall Perceived Risks versus Benefits’ section below), and 2) evaluating respondents' perceptions for 14 different nano-applications using a 4-point *risk* scale (detailed in the ‘Differences in Risk Perceptions of Nanotechnology Scenarios’ section below).

#### Differences in Overall Perceived Risks versus Benefits by Expert Group

**Figure 1:**
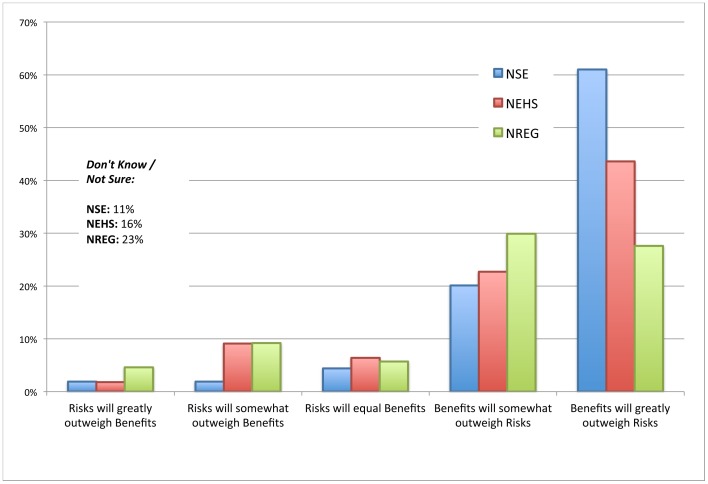
“Risk versus Benefit” ratings for nanotechnologies in general. Color-coded bars indicate the proportion of respondents in each expert group (NSE, NEHS, and NREG) choosing the indicated response.

To evaluate experts' perceptions of *risks versus benefits* of nanotechnology in general, participants were asked: “*Overall, do you think that: ‘1 - the risks of nanotechnology will greatly outweigh its benefits’, ‘2 - risks will somewhat outweigh its benefits’, ‘3 - risks will equal its benefits’, ‘4 - the benefits of nanotechnology will somewhat outweigh its risks’, ‘5 - benefits will greatly outweigh its risks’*. Respondents were also given the option to choose ‘*don’t know/not sure*’. **Figure 1** provides a summary of the results across expert groups. All three groups see *benefits* as *somewhat* or *greatly* outweighing risks, while for a small minority *risks equal* or *outweigh* benefits. Respondents from the nano scientists and engineers (NSE) group most strongly support the stance that *benefits somewhat* or *greatly* outweigh risks (NSE – 81%, NEHS – 66%, NREG – 58%). The largest difference between groups is observed for the ‘benefits *greatly* exceed risks’ response chosen by 61% of the NSE group, compared to 44% for the nano-EHS scientists and toxicologists (NEHS) group, and 28% for the scientists and regulators in government agencies (NREG) group.

Strikingly, the highest rate of ‘don't know’ responses came from the NREG group at 23%, followed by NEHS at 16% and NSE at 11%. Based on the results of a chi-square test, there is a statistically significant relationship between ‘don't know’ responses and expert group, χ^2^ (2, N = 356) = 6.611, p = .037. Taken as a proxy measure for confidence in their judgment, this indicates that NREG respondents are more hesitant to make a judgment than their counterparts when evaluating risks versus benefits. In summary, NSE respondents as a group view that benefits predominantly outweigh risks, demonstrate great confidence in their stance, and have relatively few undecided responses. Fewer experts whose research focuses on the risk implications of nanotechnologies (NEHS, NREG) demonstrate the combination of high benefit to risk ratio and low rate of ‘don't know’ responses.

We calculated mean ‘risk vs benefit’ scores for each expert group (where 1  =  risks greatly outweigh benefits, and 5  =  benefits greatly outweigh risks) and conducted a one-way ANOVA, followed by Levene's test of homogeneity of variance. The Welch F-ratio was used to test significance since variances were non-homogeneous. We found a significant difference in ‘risk vs benefit’ scores between groups (F(2, 298) = 9.76, p .001). “Don't know” responses were excluded from the analysis. A Games Howell *post hoc* analysis revealed that the ‘risk vs benefit’ score was significantly lower for both NEHS (N = 92, M = 4.16, SD = 1.1; p = .021) and NREG (N = 67, M = 3.87, SD = 1.2; p<.001) groups than for NSE (N = 142, M = 4.53, SD = 0.86). However, there was no statistically significant difference between NEHS and NREG groups (p = .252). This result partially supports our first hypothesis that perceptions of risks and benefits might differ significantly across groups, though no significant difference was found between the NEHS and NREG groups.

#### Differences in Risk Perceptions of Nanotechnology Scenarios by Expert Group

**Figure 2:**
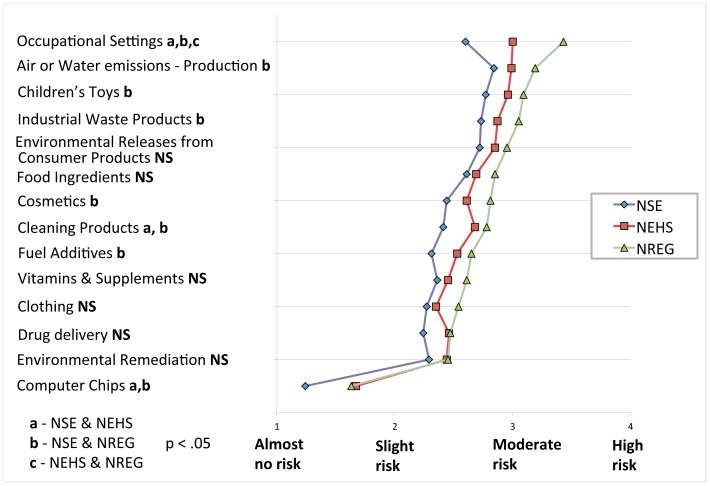
'Risk Perception' ratings for NSE, NEHS, and NREG expert groups. Mean scores for each group are indicated with points on respective color-coded lines capturing 14 different nanotechnology scenarios rated between ‘almost no risk’ and ‘high risk’. Significant differences in means were determined using a one-way ANOVA with post hoc analysis, and are indicated with a, b, and c markings as outlined in the legend.

Comparing experts across multiple nanotechnology applications was achieved by asking study participants to rate the risks of 14 nanotechnology scenarios using the following question: “*From the following list of nanomaterial applications and situations, please indicate whether you think they pose almost no risk, slight risk, moderate risk, or high risk to society*”. This four-point scale indexes ‘1’ as ‘almost no risk’ through ‘4’ as ‘high risk’; also provided was the option: ‘don’t know/not sure’. These scenarios include described situations in which nanomaterials may be encountered (e.g., in occupational settings) or released (e.g., in air or water emissions during production), and applications such as nanomaterial use in cosmetics or fuel additives. A full description of each scenario can be found in Table S1. **Figure 2** illustrates the results for the fourteen scenarios, where points on color-coded lines indicate the mean risk score for each expert group (NSE, NEHS, and NREG).

We find small but consistent differences in risk judgments between expert groups for a majority of scenarios, and a uniform trend in risk ratings across scenarios, creating roughly parallel response patterns for each group. The similarity in relative ratings of scenarios suggests a high degree of agreement between expert groups over the risk posed by one scenario relative to another. However the data illustrate that the NSE respondents perceive less risk for each scenario, while NREG respondents see the most risk, with NEHS respondents in the middle. This finding illustrates clear differences in risk perceptions between groups, which is most pronounced for the case of nanomaterials in occupational settings. Nanomaterial based computer chips receive the lowest risk rating of all scenarios.

To confirm that the observed differences in risk perceptions were significant across all 14 scenarios, we conducted a one-way between subjects Analysis of Variance (ANOVA). In this analysis, each of the fourteen scenarios was used as a dependent variable with expert group (NSE vs NEHS vs NREG) as the independent factor, followed by Levene's test of homogeneity of variance. We found that the assumption of homogeneity of variances of groups was maintained for 12 of 14 scenarios, and a separate Welch test was conducted in place of the ANOVA test for the two scenario variables with non-homogeneous variances. ANOVA and Welch test results indicated significant differences in means at the *p*<*0.05* level for 9 of 14 scenarios. A Games-Howell post hoc analysis was then conducted, with significant differences found between NSE and NREG groups on 8 of 14 scenarios, between NSE and NEHS on three scenarios, and between NEHS and NREG on just one scenario, as indicated in **Figure 2**. For complete results see Tables S1 and S2 in the Supporting Information. An additional one-way ANOVA test found no significant difference in risk perceptions between the US and Canadian respondents for each of the 14 nanotechnology scenarios. All remaining analyses performed use an aggregated sample of Canadian and US respondents within each expert category.

#### Differences in Composite Nano Risk Index by Expert Group

To determine whether the difference in means by expert group was still significant when considering all 14 nanotechnology scenarios together, we created a composite index (*hereafter referred to as ‘Nano Risk Index’*) using a principal component analysis (PCA) with orthogonal rotation (varimax). Based on a scree plot (highest Eigenvalues being 6.55, 1.35, 0.93, 0.83) we confirmed that one component accounting for 47% of the variance was adequate to explain the correlations across the 14 nanotechnology scenarios. Cronbach's alpha (α = 0.92) is evidence that the scale is internally consistent and highly reliable. Nano Risk Index factor scores were calculated using the Anderson-Rubin method, producing scores with an overall mean of zero and standard deviation of 1. Using a one-way ANOVA test we found a statistically significant difference in mean Nano Risk Index scores between groups (F(2, 401) = 9.166, p<.0001). A Tukey HSD *post hoc* analysis revealed that the Nano Risk Index score was significantly higher for both NEHS (N = 121, M = 0.07, SD = 0.97; p = .03) and NREG (N = 103, M = 0.26, SD = 0.90; p<.001) groups than for NSE (N = 180, -M = 0.20, SD = 0.84). However, there was no statistically significant difference between NEHS and NREG groups (p = .255). This result partially supports the hypothesis that risk perceptions differ significantly between NSE and NEHS groups and between NSE and NREG groups. However, our hypothesis was not supported regarding the difference in risk perceptions between NEHS and NREG groups.

### Drivers of Perceived Risks

#### Novelty, Regulatory Preferences, and Technology Risk Indices

To facilitate hypothesis testing and analysis we developed three indices based on survey responses: ‘Perceived Novelty of Benefits and Risks’; ‘Perceived Technology Risks’; and ‘Preferences for Regulation’. To test hypothesis 2, that a) experts who see *benefits as novel* will perceive less risk, and that b) experts who see *risks as novel* will perceive more risk from nanotechnologies, we developed composite indices based on a series of survey questions measuring seven dimensions of novelty. A principal component analysis (PCA) was performed with orthogonal rotation (varimax). Based on a scree plot (highest Eigenvalues being 2.24, 1.45, 0.96, 0.81) we found that two components accounting for 53% of the variance explained the correlations between seven dimensions of novelty (shown in **Table 2**). These components are measured as ‘New and Uncertain Risks‘ (5 items, α = 0.65)’ and ‘Novel Benefits and Properties’ (2 items, α = 0.74). The Anderson-Rubin method was used to calculate orthogonal factor scores with a mean of 0 and standard deviation of 1. The component ‘New and Uncertain Risks’ is moderately correlated with Nano Risk Index (Pearson's r = .39), indicating its potential as a predictor of risk perceptions, while ‘Novel Benefits and Properties’ is not meaningfully correlated (r = −.01). Both factors were included in the regression analysis below to examine their influence on nanotechnology risk perceptions.

**Table 2:** Loadings from a principal components analysis over seven rating scales averaged across individuals (VARIMAX rotated solution).

Rating Scale	*Factor 1*: New and Uncertain Risks *(31.9% of var.)*	*Factor 2*: Novel Benefits and Properties *(20.8% of var.)*
New Benefits^1^	.10	**.87**
Novel Properties^2^	.08	**.87**
Properties Cannot be Anticipated^3^ *	**.54**	.17
New Risks^4^	**.56**	.24
Risks are Not Well Known^5^ *	**.76**	−.16
Risks Cannot be Determined^6^ *	**.73**	−.02
More Uncertainty^7^	**.56**	.16

* Items are reverse coded to facilitate comparison.

*Notes: Loadings exceeding 0.4 are in boldface.*

*For each novelty question, the following Likert scale was used: 1 – Strongly Disagree, 2 – Disagree, 3 – Agree, 4 – Strongly Agree.*

1 *Nano-scale materials promise benefits for society that are not possible with bulk (non nano-scale) materials.*

2 *Nano-scale materials possess novel properties that are not expressed in their corresponding bulk forms.*

3 *The novel properties of nano-scale materials cannot be anticipated by knowing the properties of the same material in its bulk form.*

4 *Nano-scale materials pose risks for society that are not present with bulk (non nano-scale) materials.*

5 *The health and environmental risks from nano-scale materials are not well known to scientists.*

6 *The existing methods for assessing health and environmental risks from bulk materials are not suitable for determining risks from nano-scale materials.*

7 *There is more uncertainty about the risks from nano-scale materials than the risks from bulk forms.*

To test hypothesis 3: that experts' perceptions of risks from technologies in general influence their perceptions of risk for nanotechnologies specifically, we developed a comprehensive technology risk index (*hereafter referred to as Tech Risk Index*). Respondents were presented with 10 technologies commonly investigated in the risk perceptions literature, and asked to rate each scenario on the following scale: 1 – Almost No Risk, 2 – Slight Risk, 3 – Moderate Risk, 4 – High Risk. Technology scenarios consisted of *GM crops, cell phone communications, nuclear power plants, food additives and preservatives, prescription drugs, pesticides and herbicides, biofuels, vaccines, lead in paint or dust, and non-prescription vitamins and supplements*. A principal component analysis (PCA) with orthogonal (varimax) rotation was performed with all ten scenarios. Based on a scree plot (highest Eigenvalues being 3.23, 1.37, 1.00, 0.97) we found that one component explained correlations between all ten scenarios, accounting for 30% of the variance. Cronbach's alpha suggests a reliable scale (α = 0.74). Tech Risk Index scores were calculated using the Anderson-Rubin method. The Tech Risk Index is moderately correlated with risk perceptions (Pearson's r = .48), and so was also included in the regression. Given that the Tech Risk Index measures risk perceptions across a comprehensive set of technologies, we expect the index to provide a baseline measure of an expert's perceptions of technology risks.

Testing the hypothesis (4) that regulatory preferences will influence perceived risk, we developed composite indices based on a series of survey questions related to the ‘regulation of risks’ and ‘regulation of nanotechnology’, as shown in **Table 3** **.** Responses were measured using a four point Likert scale: 1 – Strongly Disagree, 2 – Disagree, 3 – Agree, 4 – Strongly Agree. A principal component analysis (PCA) of the aggregated data for these thirteen measures of attitudes toward regulation was conducted with orthogonal rotation (varimax). Based on a scree plot (highest Eigenvalues being 4.38, 1.59, 0.96, 0.93) we concluded that two orthogonal components were necessary to explain the correlations among the thirteen variables, accounting for 51% of the variance. The first component of the rotated factor loadings shown in **Table 3** is highly correlated with scales indicating that current regulations are sufficient, and indicating confidence in voluntary and market-based approaches to regulation. This factor is labeled “Confidence in Markets and Voluntary regulation” (α = 0.81). The second component is associated with the perception of inadequacy of current regulations, and preference for a precautionary approach to regulation. This factor is labeled “Preference for Precaution” (α = 0.79). Index scores were calculated using the Anderson-Rubin method. Both ‘Preference for Precaution’ (r = .43) and ‘Confidence in Markets and Voluntary Regulation’ (r = −.18) are correlated with Nano Risk Index, and were included in the regression analysis below.

**Table 3:** Loadings from a principal components analysis over fourteen rating scales related to 'Regulation of Risks' and 'Regulation of Nanotechnologies', averaged across individuals (VARIMAX rotated solution).

Rating Scale	Factor 1: Confidence in Markets and Voluntary Regulation *(33.7% of var.)*	Factor 2: Preference for Precaution *(12.2% of var.)*
***Regulation of Risks***		
The government should err on the side of precaution to protect the public from the risks from technology	−.21	**.66**
Regulations unduly prevent society from reaping the benefits of technology	**.42**	−.33
Chemical risks are sufficiently regulated in this country	**.61**	−.29
Voluntary approaches for risk management are effective for protecting human health and the environment.	**.73**	−.16
Market-based approaches are an effective means of managing health and environmental risks from technology	**.69**	−.08
Consumers should be provided with more product information to allow them to better understand a product's risks and benefits	.01	**.69**
Traditional government regulation too frequently determines that a product is dangerous when it is really safe.	.29	**−.53**
***Regulation of Nanotechnology***		
Because current regulations do not take into account novel (size-dependent) properties of nano-scale materials, they are inadequate for protecting society from risks	−.29	**.60**
Government should restrict commercial development of nanotechnology until studies have been done on how to control risks	−.12	**.74**
Companies utilizing nano-materials in their products should be required to perform more stringent toxicity testing for the products they create	−.07	**.64**
Consumers, through their purchasing decisions, are able to avoid products containing nano-scale materials if they deem them to be too risky	**.65**	.07
Government regulations, as they currently exist, will do a good job of managing risks across the entire life-cycle of nanomaterials (from initial production to end-of-life)	**.60**	−.37
Government should focus on developing voluntary programs rather than mandatory programs to manage risks from nanotechnology	**.70**	−.20

*Note: Loadings exceeding 0.4 are in boldface.*

#### Factors Influencing Experts' Perceptions of Nanotechnology Risks

The relationship between each independent variable and the dependent variable ‘Nano Risk Index’ was investigated using a hierarchical ordinary least squares (OLS) multivariate regression, shown in **Table 4**. Variables were entered in six blocks. Steps 1 through 3 introduce ‘expert group’ variables along with commonly measured demographic and domain of expertise control variables. Steps 4 through 6 introduce the ‘nanotechnology novelty’, ‘attitudes toward regulation’, and ‘Tech Risk’ index variables respectively. Other variables including ‘proportion of time working on nanotechnology’, and ‘involvement in research’, as well as ‘social and political values’, were evaluated but ultimately omitted due to non-significance in the final model. ‘Trust in government agencies’ was also tested and found to be not significant, but was a key finding in another paper [35]. Diagnostics indicate no evidence of multicollinearity (VIF<10), and that none of the four principal assumptions for linear regressions have been violated [36].

**Table 4:** Hierarchical regression with Nano Risk Index as dependent variable.

	I	II	III	IV	V	VI
*Group*						
DNEHS^a^	0.14*	0.08	0.07	0.03	0.03	0.02
DNREG	0.22***	0.18**	0.06	0.02	0.00	0.04
*Demographics*						
Gender^b^		0.16**	0.15**	0.12*	0.08	0.02
Education^c^		0.00	0.05	0.08	0.04	0.04
Year of Degree^d^		0.11*	0.10*	0.08	0.08	0.09*
*Domain of Expertise*						
Disciplinary Field^e^			0.16*	0.13	0.06	0.07
Affiliation *(Academic vs Government)* f			0.00	0.01	0.01	0.01
Affiliation *(Academic vs Other)*			−0.06	−0.02	0.03	0.00
*Nanotechnology Novelty*						
Novelty: New and Uncertain Risks^g^				0.33***	0.2***	0.21***
Novelty: Novel Benefits and Properties^h^				0.00	0.01	0.04
*Attitudes Toward Regulation*						
Regulation: Market-Based, Voluntary^i^					−0.10*	−0.10*
Regulation: Precaution^j^					0.33***	0.19***
*Technological Risk*						
Tech Risk Index^k^						0.41***
*Incremental R^2^ (%)*		3.5%***	0.5%	9.0%***	7.7%***	14.7%***
***Total R^2^ (%)***	**3.9%**	**7.4%**	**7.9%**	**16.9%**	**24.6%**	**39.3%**

*p<.05.

**p<.01.

***p<.001.

*Notes: N = 404. Independent variables were entered in six steps, where I through VI indicate model steps, and cell entries are standardized (β) regression coefficients.*

a *Paired dummy variables, where ‘NSE’ is coded as DNEHS = 0, DNREG = 0, ‘NEHS’ is coded as DNEHS = 1, DNREG = 0, and ‘NREG’ is coded as DNEHS = 0, DNREG = 1.*

b *1 = female, 0 = male.*

c *1 = PhD, 0 = Bachelors/Masters.*

d *Standardized continuous variable.*

e *1 = physical sciences, 0 = other, where ‘physical sciences’ includes chemistry, physics, materials science, chemical engineering, electrical engineering, and mechanical engineering.*

f *Paired dummy variables, where ‘academic vs government’ is coded as academic  =  0, government = 1, and ‘academic vs other’ is coded as academic = 0, other = 1.*

g–k *Continuous index variables, described above.*

We found that the perception that risks are ‘new and uncertain’ had a significant positive regression weight (*β* = 0.21, p<.001) in our final model (step VI), after controlling for the effects of demographics (gender, education, year of degree) and domain of expertise (disciplinary field and affiliation). This indicates that those individuals who perceive that risks from nanotechnologies are new and dissimilar to risks from bulk (non-nano) materials, and who perceive greater uncertainty and less ability to anticipate risks given available risk assessment methods, also see more risk overall. We also found that both ‘preference for regulatory precaution’ and Tech Risk Index had significant positive regression weights (*β* = 0.19, p<.001 and *β* = 0.41, p<.001 respectively) in the final model. This suggests that those who see more risk from other technologies, and who prefer precautionary approaches to risk management also perceive greater risks from nanotechnologies. Risk perceptions were however negatively associated with the measure of confidence in market-based and voluntary approaches for regulation (*β* = −0.10, p<.05). This finding suggests that those with greater confidence in voluntary programs and market-based approaches for managing risk also perceive less overall risk. The measure of perceived ‘novelty of benefits and properties’ was not significant. The year of graduation for participants' most recent degree also explains a small proportion of variance in the model, where more recent graduates perceive greater risk. Included as a proxy for participant's on-the-job experience, this finding suggests that younger, less experienced participants see more risk from nanotechnologies than older, more experienced participants. However the contribution to the model is small in comparison to the comprehensive index variables. Overall the model fit is good with R^2^ = 39%.

Considering the contribution of the ‘expert group’ variables (NSE, NEHS, NREG) in the regression model, their descriptive power diminishes and becomes statistically insignificant once the demographic and domain of expertise variables are entered in steps II and III. The variance explained by the ‘NSE vs NEHS’ component of the dummy variable pair (indicating the distinction between the NSE and NEHS groups) becomes insignificant with the addition of the demographic variables in step II, while ‘NSE vs NREG’ drops below the p<.05 level with the addition of the domain of expertise variables in step III. Further, ‘expert group’ variables account for just 4% of the variance in the model, with the domain and demographics variables similarly contributing only 4%. This regression analysis therefore suggests that the mean differences between groups observed in the ‘Expert Variation in Perceived Risks and Benefits’ section above are better explained by the perceptions and attitude characteristics of individuals within each expert group than by group distinction itself.

These findings support our hypotheses that experts' perceptions of the novelty of risks, perceptions of risk from other technologies, and attitudes toward regulation constitute distinct factors affecting perceptions of nanotechnology risks. We find that together these factors diminish the power of group, domain of expertise, gender, and education variables in describing observed nanotechnology risk perceptions. However, our hypothesis that perceived novelty of benefits would decrease perceived risk was rejected.

#### Novelty, Precaution, and Voluntary regulation as Characteristics of Expert groups

**Figure 3:**
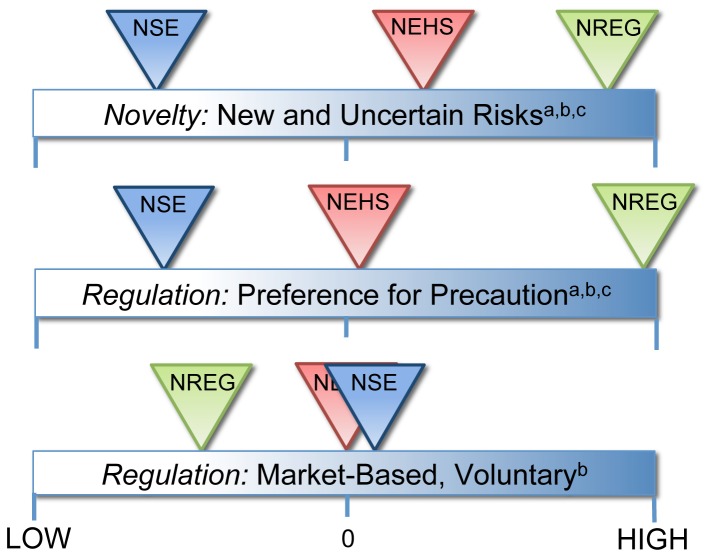
Mean scores for the 'Novelty' and 'Attitudes toward Regulation' indices for NSE, NEHS, and NREG groups. The continuum from ‘high’ to ‘low’ represents a factor score range of +/− 0.5, representing one half standard deviation in either direction from the index. a, b, and c markings indicate significant differences between groups, where a: NSE and NEHS, b: NSE and NREG, c: NEHS and NREG. Tukey HSD post hoc analysis confirms that differences in index scores are significant across all three groups for ‘Novelty’ (p<.05; NSE: N = 180, M = −0.29, SD = 0.86, NREG: N = 103, M = 0.39, SD = 0.88, NEHS: N = 121, M = 0.11, SD = 0.85), and for ‘Regulation: Preference for Precaution’ (p<.001; NSE: N = 180, M = −0.29, SD = 0.82; NEHS: N = 121, M = 0.06, SD = 0.93; NREG: N = 103, M = 0.43, SD = 0.81). Post hoc analysis confirmed a significant difference between NSE and NREG groups only for ‘Regulation: Market-Based, Voluntary’ (p<.022; NSE: N = 180, M = −0.08, SD = 0.80; NREG: N = 103, M = −0.21, SD = 0.91).

To further characterize the link between observed differences in risk perceptions by expert groups and the independent index variables explored above (new and uncertain risks, preference for precaution, preference for voluntary regulation), we calculated and compared mean index scores for each expert group, as illustrated in **Figure 3**. This figure represents the *relative difference* between groups for each index, rather than absolute scores on the Likert ‘agreement’ scale. ‘High’ and ‘Low’ scores on this scale are defined as index scores of +/− 0.5, representing one half standard deviation from the index mean for the Anderson-Rubin calculated indices. Here we see for the index ‘*Novelty: New and Uncertain Risks*’ that the NSE group on average scores the lowest, while the NREG group scores the highest. The NEHS group is also above the mean score for the index. A One-Way ANOVA analysis confirms that the observed difference in means is significant (F(2, 401) = 22.17, p<.001), and a Tukey HSD *post hoc* analysis confirms that mean scores are significantly different across all three groups. This indicates a larger difference in perceptions of the novelty of nanotechnology risks between the NSE and NREG groups, than between NSE and NEHS groups.

For the ‘*Regulation: Preference for Precaution*’ index in **Figure 3** we see a pattern similar to the novelty index with NREG scoring highest on the index, NSE on the opposite end of the spectrum, and NEHS roughly at the center point. A One-Way ANOVA confirms that the mean scores are significantly different (F(2, 401) = 24.23, p<.001), and a Tukey HSD *post hoc* analysis confirms significant differences between all three groups. As a whole, NREG respondents most strongly prefer precautionary approaches to regulation, while NSE respondents prefer precaution the least. For the ‘*Regulation: Market-Based, Voluntary*’ index, the NSE and NEHS groups reflect the average score for the index, while NREG indicates relatively less support for current regulations and market-based or voluntary approaches. A One-Way ANOVA confirms that the mean scores are significantly different (F(2, 401) = 3.89, p<.001), and a Tukey HSD *post hoc* analysis confirms significant differences between the NSE and NREG group only.

**Figure 4:**
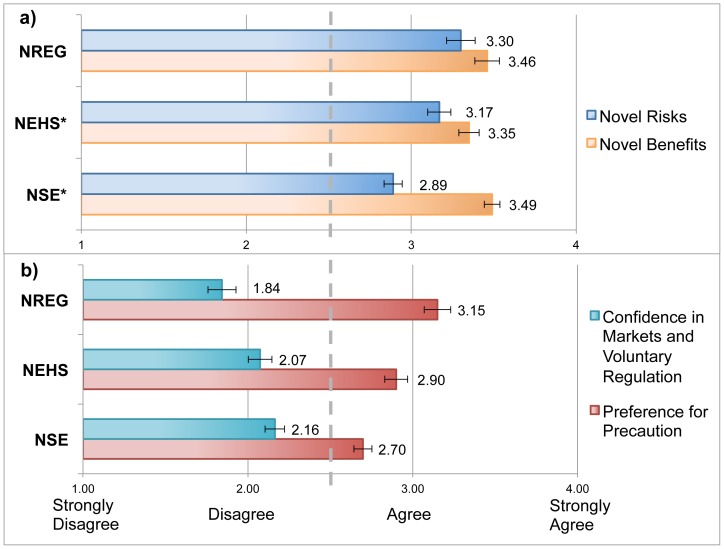
Comparison of perceptions of ‘novelty’ and ‘attitudes towards regulation’ across expert groups: *a)* Perceptions of the novelty of benefits versus novelty of risks. *b)* ‘Confidence in Markets and Voluntary Regulation’ versus ‘Preference for Precaution’. * indicates significant difference in means between ‘novel risks’ and ‘novel benefits’ by paired t-test, where Novel Benefits M = 3.50, SD = 0.58, Novel Risks M = 2.89, SD = 0.65 for NSE group; Novel Benefits M = 3.3, SD = 0.62, Novel Risks M = 3.16, SD = 0.67, for NEHS group; and difference in means for NREG group is not significant.

To evaluate scores in absolute terms, we compared responses for several survey questions based on the Likert ‘agreement’ scale. For the individual ‘novelty’ survey items, we compared two questions to gauge the difference in agreement between groups on the *novelty of benefits* and the *novelty of risks*. Participants were asked to answer the following questions using a four point likert agreement scale: i) Novel Benefits: “*Nano-scale materials promise benefits for society that are not possible with bulk (non nano-scale) materials*”; and ii) Novel Risks: “*Nano-scale materials pose risks for society that are not present with bulk (non nano-scale) materials*”. **Figure 4** **a**) shows that while all three groups on average agree that nanotechnologies pose both novel benefits and novel risks (mean scores are greater than 2.5), there is a consistent difference in agreement between these two items across groups, where risks are seen as *less novel* than are the benefits. This difference in novelty perceptions is most pronounced for the NSE group, where a paired t-test finds a significant difference of 0.61 between ‘novel benefits’ and ‘novel risks’ (t(140) = 8.59, p<.001) compared to 0.17 for the NEHS group (t(90) = 2.06, p = .042) and 0.14 for the NREG group (t(69) = 1.52, p = .133, not significant). NSE respondents on average see far less ‘novel risk’ from nanotechnologies, yet view a similar level of ‘novel benefits’ compared to other groups.

For the *attitudes towards regulation* indices, absolute scores were calculated by averaging responses across survey items for each of factors 1 and 2 (listed in **Table 3** above) to provide scores on the 4-point ‘agreement’ scale. **Figure 4b** **)** shows that the mean score for each group is less than 2.5 for the ‘Confidence in Markets and Voluntary Regulation’ index, indicating overall disagreement with questions on the sufficiency of current regulations and support for market-based or voluntary approaches to regulation. However, NREG respondents disagree most strongly compared to the NEHS and NSE groups. Conversely, average ‘*Preference for Precaution*’ scores indicated agreement with questions related to precautionary approaches to regulation, and greater restriction of nanotechnology development.

**Figure 5:**
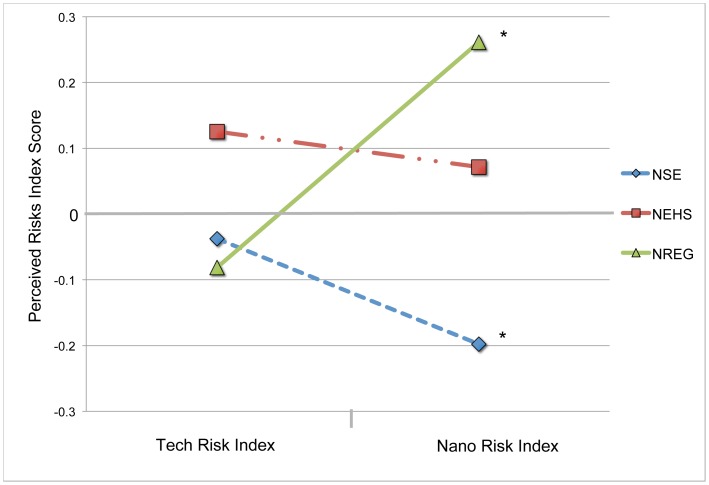
Comparison of Tech Risk Index and Nano Risk Index scores by expert group. Paired t-test scores confirmed a significant difference in means between Tech Risk Index and Nano Risk Index for the both the NREG group (Tech Risk Index M = −0.08, SD = 0.99; Nano Risk Index M = 0.26, SD = 0.90), and for the NSE group (Tech Risk Index M = −0.04, SD = 0.82; Nano Risk Index M = −0.20, SD = 0.84). * indicates significant difference in means between Tech Risk Index and Nano Risk Index scores.

In order to compare risk perceptions between nanotechnology risk scenarios and (non-nano) technology risk scenarios, we compared Tech Risk Index and Nano Risk Index scores in **Figure 5**. Here we see that all three expert groups score at or near the mean Tech Risk Index score. A One-Way ANOVA finds no significant difference in means between groups for Tech Risk Index. However, the mean Nano Risk Index scores were found to differ significantly between NSE and the NREG and NEHS groups as previously described in the ‘Differences in Composite Nano Risk Index’ section above. In terms of *within group* differences, we see that the mean Nano Risk Index score is greater than the mean Tech Risk Index score for the NREG group by 0.34. A paired t-test confirmed that the difference in mean scores is significant (t(102) = 3.822, p<.001). Conversely, the mean Nano Risk Index score for the NSE group was found to be significantly less by paired t-test than the Tech Risk Index score (M = −0.16; t(179) = −2.53, p = .012). The mean Nano Risk Index score for the NEHS group was slightly lower but not significantly different than the corresponding Tech Risk Index score. This finding suggests that those in the NREG group see nanotechnology risks differently than the other groups, perceiving greater risk from nanotechnologies than other technologies compared to the NSE and NEHS groups who see less.

## Discussion

Our observation that experts' perceptions of risk from nanotechnologies differ significantly across domains of expertise holds true between both the NSE and NEHS, and the NSE and NREG groups. Nanoscientists and engineers at the upstream end of the life cycle were found to perceive significantly less risk from nanotechnologies compared to those who are responsible for the downstream assessment and regulation. These results partially support our first hypothesis: that nanotechnology risk perceptions will differ between groups. We did not however find a significant difference between NEHS and NREG groups on this measure. By further characterizing the attitudes that define each expert group, our analysis revealed significant differences between groups given three discrete indices: perceptions of the novelty of risks, preference for precaution, and confidence in market and voluntary regulation. Nanotechnologies were also perceived differently compared to other non-nano technologies for the NSE and NREG groups, albeit with opposite trends.

### Characteristic Differences in Expert Group Perceptions and Attitudes

Considering the four composite indices (*novelty, precaution, market-based/voluntary regulation, technology risk*) tested here, the observed differences in mean index scores provide insight into the characteristic attitudes of each expert group. NSE respondents viewed nanotechnologies to pose significantly less risk than other technologies. NSE respondents also scored the *lowest* on *precaution* and *novelty* of risks on average, corresponding with their low mean Nano Risk Index scores. This mirrors the insights provided by Powell's interviews with scientists [5] and Harthorn & Bryant's [32] focus group work. Both of these early studies posited, but did not quantitatively test, the fact that NSE experts more frequently express reservations when nanotechnologies are viewed as being new or different than other technologies or materials in their bulk form. Our research confirms these early suggestions of the importance of perceived novelty. We also find a sizable disparity between ‘Novelty of Benefits’ versus ‘Novelty of Risks’ judgments (**Figure 4a**) for the NSE group, which indicates that benefits are seen as “new”, yet risks are “not” or at least, much less so. These findings together strongly suggest that NSE experts are more optimistic in their views (this is similar to what is referred to as an ‘optimism bias’ [37] in the risk perceptions literature).

The finding that nanotechnology benefits will *strongly outweigh risks* among NSE respondents (with relatively few undecided in their response) further supports the idea that optimism is pervasive amongst NSE experts. This is not necessarily surprising given their close proximity to the design and development of these technologies (i.e., at the ‘upstream’ end of the nanotechnology life-cycle), a process often engendered by the hope of new and beneficial applications. Finally, NSE respondents also demonstrated the greatest support among groups for a hands-off, free-market approach to managing nanotechnology risks. This suggests that, consistent with their optimism, NSE experts are also more likely to perceive top-down, or precautionary regulation as threatening development opportunities and their benefits.

In contrast with the NSE group, NREG respondents perceived the greatest novelty of risks, on par with their perceptions of novel benefits (**Figure 4a** **.**). They also had the lowest average among groups on the risks versus benefits rating for nanotechnologies in general, with a mean score between ‘risks will equal benefits’ and ‘benefits will somewhat outweigh risks’. This suggests that NREG respondents recognize that novel nanomaterial properties may pose *both* benefits and risks in more or less competing or equal fashion. NREG respondents on average also scored the *highest* on precaution, and the *lowest* on the market/voluntary regulation index. This trend closely tracks closely with the high Nano Risk Index score observed for the NREG group. Further, comparison of nano risk perceptions with the comprehensive technology risk index (Tech Risk Index) shows that NREG respondents on average see more risk from nanotechnologies than from other technologies, while NSE and NEHS respondents see less. Together these findings suggest that NREG respondents are more likely to see nanotechnology as new and risky, and prefer precautionary top-down regulatory approaches to manage risks rather than to leave regulation to market-based mechanisms. Hence, compared to NSE respondents, NREG respondents display a tendency towards negativity or worry with respect to nanotechnology risks and benefits. These ‘cautious regulators’ are likely highly attuned to the challenges of assessing and managing risks, directly face the challenge of regulating nanotechnologies on a day-to-day basis, and have first-hand experience with the limitations of market-based and voluntary approaches to regulation [38]. Together these experiences are likely responsible for the observed pattern of precaution, the belief that nanotechnology is new and more uncertain, and the attention to risk that is not seen with other groups.

NEHS respondents' perceptions of novelty of risk, preference for precaution, and confidence in market and voluntary regulation were found to lie consistently between the NSE and NREG groups. However, the differences between the NEHS respondents and NSE and NREG groups were only significant for their perception of the novelty of risks and preference for precautionary regulation. For perceptions of nanotechnology risk (Nano Risk Index), no difference is noted between the NEHS and NREG groups. Given the NEHS experts' focus on assessment of risks and direct experience with the use and limitations of risk assessment methodologies, it is understandable that NEHS experts would be more attuned to the limitations of risk assessment methodologies for nanotechnologies than would NSE respondents, though perhaps less so than NREG respondents.

### Perceived Novelty of Risks, Attitudes Toward Regulation, and Perceptions of Technology Risk as Drivers of Nanotechnology Risk Perceptions

The results of a multivariate regression analysis confirmed that while expertise differentiates perceived risk, that pattern did not hold in the final regression model. Rather, a large proportion of the variance was described by the four composite indices. The implications of this finding are explored below for each such comprehensive index variable.

There is continued disagreement between experts on whether or not nanotechnology is indeed a new and distinct domain of science and engineering, and whether nanotechnologies pose new or different risks than their bulk (non-nano) counterparts [5], [32]. This unresolved debate was strongly manifest in our findings. Perceived novelty of risks was a significant factor driving overall nanotechnology risk perceptions. This finding echoes similar results based on the psychometric paradigm [13], [24], [39] in which perceived *uncertainty*, and judgments of whether *risks are ‘known’*, were found to be drivers of overall risk perceptions [39]–[41]. Our ‘novelty of risk’ index explored whether experts believed that risks were *different* then conventional (non-nano) materials (and hence uncertain), whether the *uncertainty* was greater than for non-nano materials, and whether their properties can be *anticipated* by knowing the properties of the bulk (non-nano) material. In this sense, ‘Novelty: New and Uncertain Risks’ indicates an overall uncertainty in both the types of risks and magnitude of risks posed by nanotechnologies. The judgment that *current methods are not suitable* to assess these risks may reinforce an experts' sense of uncertainty, further contributing to their perceptions of risks.

We also found that perceptions of risk from other technologies, measured here with a comprehensive set of technologies frequently studied in the risk literature, proved to be a good predictor of risk perceptions for nanotechnologies. We found that experts who see more risk overall from technologies are more likely to see greater risk from nanotechnologies as well. Given the diverse set of technologies used in the creation of this index, we expect this result is robust. This approach is nonetheless a methodology worthy of further exploration in future research.

Attitudes towards regulation were assessed along two dimensions, including *preference for precaution* in regulation and *confidence in market-based and voluntary approaches* to risk management. Together these dimensions reflect a measure similar to *support for regulation,* measured by Besley et al. (2008), or *need for regulation*, by Corley et al. (2009), confirming that the expert groups studied here would prefer more government regulation as a precaution (though the NSE group scored lowest on this index, Fig 4b). However the ‘precaution’ index is a complex measure of experts' attitudes and indicates both dissatisfaction with current levels of regulation, and preference for precautionary actions including measures to restrict commercial development, to require additional testing, and to provide consumers with additional product information. The relationship between the ‘precaution’ index and nano risks perceptions (in the regression **Table 4**) demonstrates that experts' generalized attitudes toward precautionary regulation color their perceptions of risk: those with more precautionary predispositions see more overall risk than those who favor less precaution.

Like the ‘Preference for Precaution’ measure, the ‘market/voluntary regulation’ index is also reflective of experts' attitudes toward regulation in general, and their preference for less government regulation and a free-market approach. However in absolute terms (Fig 4b), this measure did not receive much overall support, with experts on average disagreeing with the survey items composing the index. This index also played a minor role in the regression, indicating little influence overall on perceived nanotechnology risks.

## Conclusions

This research shows that differences in nanotechnology risk perceptions across expert groups are not driven by the group distinction *per se*, but rather are the result of characteristic perceptions and attitudes of the experts within each group. These characteristics are reflective of where the experts are situated along the nanotechnology life cycle, their focus on creation, testing, or regulation of nanotechnologies, and their familiarity with the challenges corresponding to risk assessment and regulation. Together these factors account for the observed predispositions toward *optimism* at the upstream, generative end of the life cycle, versus *caution* at the downstream, risk-regulation end. These ‘expert group’ distinctions provide insight into the complexity of risk perceptions, opinions, and regulatory attitudes that can be expected from experts in each group. While all experts surveyed here are involved in the multidisciplinary nanotechnology enterprise, they each constitute different and distinct points of view, drawn closely from experiences in nanotechnology development, risk evaluation, and regulation. These opinions may also be reflective of predominant opinions and attitudes that derive from institutional cultures, and are a function of training, affiliation, and experience. As such these opinions may reflect optimistic attitudes such as in the NSE group, and a tendency toward caution in the NREG group. These findings reinforce the need to be aware of inherent biases and predispositions among experts from different groups, which can lead to possible attenuation or amplification of risk judgments, and can influence decisions on which (nano)-technologies deserve attention and why. Ultimately, it is important to consult experts from across the life cycle, from upstream development to downstream testing and regulation, to ensure a cross sample of opinion, and to draw upon diverse expertise in the search for appropriate approaches for managing risks. Regardless, all three expert groups believe current regulations to be insufficient for managing nanomaterial risks, and they generally support the use of precautionary approaches to regulation over market-based or voluntary ones, albeit at varying levels within each group.

## Supporting Information

Table S1 **One-Way Analysis of Variance (ANOVA) measuring significance of differences in mean Risk Perceptions by expert group for 14 nanotechnology scenarios (scale: ‘1- almost no risk’, ‘2 –slight risk’, ‘3 – moderate risk’, ‘4 – high risk’).**
(DOCX)

Table S2 **Games-Howell post hoc analysis indicating significant differences in means between NSE-NEHS, NSE-NREG, and NEHS-NREG group pairings.**
(DOCX)

List S1 **Agencies involved in NREG sample selection.**
(DOCX)
