# Supplementary material for: Scientists versus Regulators: Precaution, Novelty & Regulatory Oversight as Predictors of Perceived Risks of Engineered Nanomaterials
Source: PLoS One. 2014 Sep 15;9(9):e106365. doi: 10.1371/journal.pone.0106365 (PMC4164444; doi:10.1371/journal.pone.0106365)
Supplement: Table S1 — One-Way Analysis of Variance (ANOVA) measuring significance of differences in mean Risk Perceptions by expert group for 14 nanotechnology scenarios (scale: ‘1- almost no risk’, ‘2 –slight risk’, ‘3 – moderate risk’, ‘4 – high risk’). (DOCX) [file pone.0106365.s001.docx]

Table S1. One-Way Analysis of Variance (ANOVA) measuring significance of differences in mean Risk Perceptions by expert group for 14 nanotechnology scenarios (scale: ‘1- almost no risk’, ‘2 –slight risk’, ‘3 – moderate risk’, ‘4 – high risk’)

| **Nanotechnology Scenario** | **GROUP** | **N** | **Mean** | **S.D.** | **Levene Test for Homogeneity of Variances** | | **ANOVA** | |
| --- | --- | --- | --- | --- | --- | --- | --- | --- |
|  |  |  |  |  | **Levene Statistic** | **p-value** | **F-value** | **p-value** |
| Drug delivery via nano-capsules | NSE | 138 | 2.24 | 0.74 | 1.826 | 0.163 | 3.344 | 0.037 |
|  | NEHS | 98 | 2.46 | 0.802 |  |  |  |  |
|  | NREG | 73 | 2.47 | 0.709 |  |  |  |  |
| Nanotechnology vitamin and mineral supplements | NSE | 124 | 2.36 | 0.79 | 2.255 | 0.107 | 1.955 | 0.144 |
|  | NEHS | 92 | 2.45 | 0.918 |  |  |  |  |
|  | NREG | 67 | 2.61 | 0.778 |  |  |  |  |
| Cosmetics with nano-particle additives | NSE | 137 | 2.44 | 0.746 | 1.257 | 0.286 | 5.346 | 0.005 |
|  | NEHS | 95 | 2.61 | 0.842 |  |  |  |  |
|  | NREG | 79 | 2.81 | 0.878 |  |  |  |  |
| Nano-particle based cleaning products | NSE | 135 | 2.41 | 0.776 | 1.786 | 0.169 | 5.796 | 0.003 |
|  | NEHS | 92 | 2.68 | 0.889 |  |  |  |  |
|  | NREG | 74 | 2.78 | 0.798 |  |  |  |  |
| Nano-particles in environmental remediation (contaminated site cleanup) applications | NSE | 134 | 2.29 | 0.724 | 2.295 | 0.103 | 1.453 | 0.236 |
|  | NEHS | 95 | 2.44 | 0.808 |  |  |  |  |
|  | NREG | 76 | 2.45 | 0.839 |  |  |  |  |
| Nano-based food ingredients | NSE | 133 | 2.61 | 0.851 | 0.957 | 0.385 | 1.868 | 0.156 |
|  | NEHS | 87 | 2.69 | 0.88 |  |  |  |  |
|  | NREG | 74 | 2.85 | 0.871 |  |  |  |  |
| Nano-particles released to the environment (air, water, soil) from consumer products | NSE | 141 | 2.72 | 0.848 | 1.932 | 0.147 | 2.191 | 0.113 |
|  | NEHS | 96 | 2.85 | 0.781 |  |  |  |  |
|  | NREG | 79 | 2.95 | 0.815 |  |  |  |  |
| Nano-particle coating on children’s toys | NSE | 130 | 2.77 | 0.885 | 0.732 | 0.482 | 3.473 | 0.032 |
|  | NEHS | 95 | 2.96 | 0.874 |  |  |  |  |
|  | NREG | 75 | 3.09 | 0.857 |  |  |  |  |
| Nano-particles as fuel additives | NSE | 140 | 2.31 | 0.856 | 1.159 | 0.315 | 4.13 | 0.017 |
|  | NEHS | 91 | 2.53 | 0.861 |  |  |  |  |
|  | NREG | 72 | 2.65 | 0.937 |  |  |  |  |
| Nanomaterials in air or water emissions from production facilities | NSE | 140 | 2.84 | 0.825 | 0.952 | 0.387 | 4.743 | 0.009 |
|  | NEHS | 97 | 2.99 | 0.784 |  |  |  |  |
|  | NREG | 79 | 3.19 | 0.786 |  |  |  |  |
| Nano-materials in occupational settings | NSE | 135 | 2.6 | 0.812 | 1.703 | 0.184 | 27.985 | 0 |
|  | NEHS | 97 | 3 | 0.829 |  |  |  |  |
|  | NREG | 80 | 3.43 | 0.689 |  |  |  |  |
| Nanomaterials in industrial waste products | NSE | 140 | 2.73 | 0.847 | 1.468 | 0.232 | 3.815 | 0.023 |
|  | NEHS | 95 | 2.87 | 0.854 |  |  |  |  |
|  | NREG | 75 | 3.05 | 0.751 |  |  |  |  |
| **Nanotechnology Scenario** | **GROUP** | **N** | **Mean** | **S.D.** | **Levene Test for Homogeneity of Variances** | | **Welch Test for equality of means** | |
|  |  |  |  |  | **Levene Statistic** | **Sig.** | **Statistic^a^** | **Sig.** |
| Nanotechnology based computer chips and devices | NSE | 150 | 1.24 | 0.514 | 23.677 | 0 | 18.18 | 0 |
|  | NEHS | 99 | 1.67 | 0.728 |  |  |  |  |
|  | NREG | 80 | 1.63 | 0.682 |  |  |  |  |
| Clothing with antibacterial nano-particle treatments | NSE | 139 | 2.27 | 0.788 | 3.173 | 0.043 | 2.587 | 0.078 |
|  | NEHS | 93 | 2.35 | 0.905 |  |  |  |  |
|  | NREG | 79 | 2.54 | 0.874 |  |  |  |  |
