# Supplementary material for: Scientists versus Regulators: Precaution, Novelty & Regulatory Oversight as Predictors of Perceived Risks of Engineered Nanomaterials
Source: PLoS One. 2014 Sep 15;9(9):e106365. doi: 10.1371/journal.pone.0106365 (PMC4164444; doi:10.1371/journal.pone.0106365)
Supplement: Table S2 — Games-Howell post hoc analysis indicating significant differences in means between NSE-NEHS, NSE-NREG, and NEHS-NREG group pairings. (DOCX) [file pone.0106365.s002.docx]

Table S2. Games-Howell post hoc analysis indicating significant differences in means between NSE-NEHS, NSE-NREG, and NEHS-NREG group pairings

| **Dependent Variable** | **(I) GROUP** | **(J) GROUP** | **Mean Difference (I-J)** | | **Std. Error** | **p-value** | **95% Confidence Interval** | |
| --- | --- | --- | --- | --- | --- | --- | --- | --- |
|  |  |  |  |  |  |  | **Lower Bound** | **Upper Bound** |
| Nanotechnology based computer chips and devices | NSE | NEHS | *-.427** | | 0.084 | 0 | -0.63 | -0.23 |
|  |  | NREG | *-.385** | | 0.087 | 0 | -0.59 | -0.18 |
|  | NEHS | NREG | 0.042 | | 0.106 | 0.918 | -0.21 | 0.29 |
| Drug delivery via nano-capsules | NSE | NEHS | -0.22 | | 0.103 | 0.084 | -0.46 | 0.02 |
|  |  | NREG | -0.227 | | 0.104 | 0.079 | -0.47 | 0.02 |
|  | NEHS | NREG | -0.007 | | 0.116 | 0.998 | -0.28 | 0.27 |
| Nanotechnology vitamin and mineral supplements | NSE | NEHS | -0.083 | | 0.119 | 0.767 | -0.36 | 0.2 |
|  |  | NREG | -0.249 | | 0.119 | 0.093 | -0.53 | 0.03 |
|  | NEHS | NREG | -0.166 | | 0.135 | 0.436 | -0.49 | 0.15 |
| Clothing with antibacterial nano-particle treatments | NSE | NEHS | -0.081 | | 0.115 | 0.759 | -0.35 | 0.19 |
|  |  | NREG | -0.271 | | 0.119 | 0.062 | -0.55 | 0.01 |
|  | NEHS | NREG | -0.189 | | 0.136 | 0.346 | -0.51 | 0.13 |
| Cosmetics with nano-particle additives | NSE | NEHS | -0.173 | | 0.107 | 0.245 | -0.43 | 0.08 |
|  |  | NREG | *-.372** | | 0.118 | 0.005 | -0.65 | -0.09 |
|  | NEHS | NREG | -0.2 | | 0.131 | 0.284 | -0.51 | 0.11 |
| Nano-particle based cleaning products | NSE | NEHS | *-0.27** | | 0.114 | 0.05 | -0.54 | 0 |
|  |  | NREG | *-.369** | | 0.114 | 0.004 | -0.64 | -0.1 |
|  | NEHS | NREG | -0.099 | | 0.131 | 0.731 | -0.41 | 0.21 |
| Nano-particles in environmental remediation (contaminated site cleanup) applications | NSE | NEHS | -0.151 | | 0.104 | 0.315 | -0.4 | 0.09 |
|  |  | NREG | -0.156 | | 0.115 | 0.364 | -0.43 | 0.12 |
|  | NEHS | NREG | -0.005 | | 0.127 | 0.999 | -0.31 | 0.3 |
| Nano-based food ingredients | NSE | NEHS | -0.081 | | 0.12 | 0.779 | -0.36 | 0.2 |
|  |  | NREG | -0.242 | | 0.125 | 0.133 | -0.54 | 0.05 |
|  | NEHS | NREG | -0.162 | | 0.138 | 0.474 | -0.49 | 0.17 |
| Nano-particles released to the environment (air, water, soil) from consumer products | NSE | NEHS | -0.138 | | 0.107 | 0.403 | -0.39 | 0.11 |
|  |  | NREG | -0.233 | | 0.116 | 0.114 | -0.51 | 0.04 |
|  | NEHS | NREG | -0.095 | | 0.121 | 0.714 | -0.38 | 0.19 |
| Nano-particle coating on children’s toys | NSE | NEHS | -0.189 | | 0.119 | 0.252 | -0.47 | 0.09 |
|  |  | NREG | *-.324** | | 0.126 | 0.029 | -0.62 | -0.03 |
|  | NEHS | NREG | -0.135 | | 0.134 | 0.569 | -0.45 | 0.18 |
| Nano-particles as fuel additives | NSE | NEHS | -0.22 | | 0.116 | 0.14 | -0.49 | 0.05 |
|  |  | NREG | *-.346** | | 0.132 | 0.026 | -0.66 | -0.03 |
|  | NEHS | NREG | -0.125 | | 0.143 | 0.655 | -0.46 | 0.21 |
| Nanomaterials in air or water emissions from production facilities | NSE | NEHS | -0.147 | | 0.106 | 0.349 | -0.4 | 0.1 |
|  |  | NREG | *-.347** | | 0.113 | 0.007 | -0.61 | -0.08 |
|  | NEHS | NREG | -0.2 | | 0.119 | 0.215 | -0.48 | 0.08 |
| Nano-materials in occupational settings | NSE | NEHS | *-.400** | | 0.109 | 0.001 | -0.66 | -0.14 |
|  |  | NREG | *-.825** | | 0.104 | 0 | -1.07 | -0.58 |
|  | NEHS | NREG | *-.425** | | 0.114 | 0.001 | -0.69 | -0.16 |
| Nanomaterials in industrial waste products | NSE | NEHS | -0.145 | | 0.113 | 0.406 | -0.41 | 0.12 |
|  |  | NREG | *-.325** | | 0.112 | 0.012 | -0.59 | -0.06 |
|  | NEHS | NREG | -0.18 | | 0.123 | 0.314 | -0.47 | 0.11 |
| *. The mean difference is significant at the 0.05 level. | | | |  |  |  |  |  |
