# Supplementary material for: Scientists versus Regulators: Precaution, Novelty & Regulatory Oversight as Predictors of Perceived Risks of Engineered Nanomaterials
Source: PLoS One. 2014 Sep 15;9(9):e106365. doi: 10.1371/journal.pone.0106365 (PMC4164444; doi:10.1371/journal.pone.0106365)
Supplement: List S1 — Agencies involved in NREG sample selection. (DOCX) [file pone.0106365.s003.docx]

**List S1. Agencies involved in NREG sample selection**

***CANADIAN FEDERAL AGENCIES^*^***

Environment Canada

Health Canada

CFIA – Canadian Food Inspection Agency

CIHR – Canadian Institutes of Health Research

DFAIT – Foreign Affairs and International Trade Canada

IC – Industry Canada

NRC – National Research Council

NRCAN – Natural Resources Canada

** due to the difficulty of identifying subjects at the provincial level in Canada, subject selection was limited to federal agencies*

***US AGENCIES***

Federal Level Regulatory Agencies:

EPA – Environmental Protection Agency – National Offices, Region 1, Region 5

FDA – US Food and Drug Administration

OSHA – US Occupational Safety and Health Administration

USDA – US Department of Agriculture

Other Federal Level (non-regulatory) Agencies, Labs, and Institutes involved in Nano risk research and Regulation:

NIH – National Institutes of Health

NIOSH – National Institute for Occupational Safety and Health

NIST – National Institutes of Standards and Technology

ANL - Argonne National Lab

BNL - Brookhaven National Lab

LANL – Los Alamos National Lab

LBL – Lawrence Berkeley Lab

LLNL – Lawrence Livermore National Lab

NCI - National Cancer Institute

ORNL - Oak Ridge National Lab

PNNL – Pacific Northwest National Lab

Air Force

Ames Laboratory

Army

Navy

NSF - National Science Foundation

State Level Agencies:

California EPA – DTSC – Department of Toxic Substances Control

Massachusetts Department of Environmental Protection

Massachusetts Department of Labor

Massachusetts Department of Public Health

Massachusetts Division of Occupational Safety

Massachusetts Office of Business Development

Massachusetts Office of Technical Assistance and Technology

North Carolina NCDENR - NC Dept. of Environment and Natural Resources

New York Department of Environmental Conservation

New York Department of Health

Washington Department of Ecology
